# Mg/Li Co-Doping Activates Anionic Redox in Sodium-Ion Battery Layered Oxides

**DOI:** 10.3390/ma19102006

**Published:** 2026-05-12

**Authors:** Wenchao Zhan, Yuefeng Wang, Xumin Wang, Hao Yang, Qianqian Feng, Xianfen Wang

**Affiliations:** Institute of Materials for Energy and Environment, College of Materials Science and Engineering, Qingdao University, Qingdao 266071, China

**Keywords:** sodium-ion battery, cathode material, doping modification, oxygen ion redox

## Abstract

Thanks to their low cost and environmental sustainability, sodium-ion batteries have emerged as a highly attractive alternative to lithium-ion systems in the field of large-scale energy storage; however, issues such as insufficient energy density and poor cycle stability have hindered their widespread adoption. We have rationally designed a Mg/Li co-doped P2-type NLMMO (Na_0.8_Mg_0.22_Li_0.08_Mn_0.7_O_2_) cathode material that enables reversible anion redox reactions through synergistic interactions, enhancing the stability of the layered framework. The material exhibits an exceptional initial discharge capacity of 158 mAh g^−1^ at 2.0–4.4 V and retains 68% of its capacity after 400 cycles at 0.1 A g^−1^, surpassing the performance of both lithium-doped (Na_0.8_Li_0.3_Mn_0.76_O_2_, NLMO) and magnesium-doped (Na_0.8_Mg_0.3_Mn_0.7_O_2_, NMMO) materials. In situ XRD shows that NLMMO has structural stability, and the Mg doping modification strongly inhibits the phase transition and stabilizes the interlayer structure. Ex situ XPS analysis indicates that the lattice oxygen in the cathode material underwent changes. This study provides a new path for designing cathodes with synergistic anionic and cationic redox reactions.

## 1. Introduction

Sodium-ion manganese-based layered transition metal oxides are known for their high theoretical specific capacity and excellent structure. P2-type manganese-based materials are currently of great interest and popularity due to their favorable conformation for sodium ion diffusion and high redox charge compensation. However, during high-voltage charging and discharging, the weakening of the electrostatic shielding of TMO_2_ due to the Jahn–Teller effect caused by the manganese ion valence change and sodium ion desodiation leads to severe irreversible distortions and slippage in the laminar structure, which destroys the sodium ion diffusion channel and causes the battery capacity to decrease [1,2]. In fact, the storage and release of energy from P2-type Mn-base cathode materials is achieved by redox reactions of transition metal ions (Ni, Mn, Co, Fe) [3,4,5].

Replacing Mn with non-redox-active elements (Li, Mg, Zn, Ca) in the transition metal layer (TM) can simultaneously achieve a reduction in the number of Mn^3+^ Jahn–Teller centers and suppression of phase transitions during charging and discharging, which can lead to an improvement in the cathode material properties [6,7,8,9,10,11,12].

Severe phase transitions and structural slips occur, resulting in transition metal dissolution and other side reactions at the interface side of the electrode. Consequently, interfacial engineering and electrolyte modification are recognized as effective methods to enhance sodium-ion manganese-based materials [13,14,15,16,17,18]. Currently, there is a consensus that the addition of organic substances capable of forming a dense CEI film on the cathode material, or the application of a dense inorganic coating directly on the cathode material surface, is a viable approach [19,20,21,22,23]. The use of solid-state electrolytes is also an effective means of improving the interface [24,25].

The issue of low energy density in P2-type materials is a significant challenge that hinders the application of manganese-based oxide materials. Recent studies have shown that batteries can achieve higher energy densities when oxygen is involved in charge compensation [26,27]. The doping of the transition metal layer with elements such as Li, Mg, and Zr has been shown to mobilize anion redox. Li substitution of Mn in the transition metal layer has been shown to stimulate the redox reactions of neighboring oxygen atoms. The generation of the Na-O-Li conformation in the microstructure has been demonstrated to result in a non-hybrid O_2p_ state, which allows for anionic redox and gives the cathode material a capacity boost [7,28].

Zr doping to form a stable monolithic structure can inhibit oxygen generation. The principle is that Zr can penetrate the AM (alkali metal layer) and nail into place to prevent interlayer slippage. In addition, the Zr-O conformation can resist electrolyte erosion during cycling, which improves cycling stability [29,30]. Mg doping in the TM layer suppresses oxygen generation and prevents irreversible phase transitions because Mg^2+^ in a deeply dissociated condition can interact with under-coordinated and electron-deficient O_2p_, thus suppressing oxygen generation [8,29,31,32].

In this work, co-doped P2-Na_0.8_Li_0.08_Mg_0.22_Mn_0.7_O_2_ (NLMMO) cathodes are synthesized via the Li/Mg co-substitution strategy. Na_0.8_Li_0.3_Mn_0.76_O_2_ (NLMO) and Na_0.8_Mg_0.3_Mn_0.7_O_2_ (NMMO) are prepared for comparison. It is found that NLMO can effectively stimulate oxygen redox with improved capacity and that NMMO has a very stable long cycle and good rate performance. The Li/Mg co-doped NLMMO retains both of these advantages simultaneously. In particular, NLMMO achieves a capacity of 158 mAh g^−1^ at 2–4.4 V in the first cycle and a capacity retention of 68% after 400 cycles at a current density of 0.1 A g^−1^. Combined in situ XRD and ex situ XPS revealed the microstructural changes and redox mechanism of NLMMO. The electrochemical performance of NLMMO suggests that the P2-type layered oxide material is a promising cathode candidate for practical applications.

## 2. Experimental Section

### 2.1. Materials Synthesis

NLMMO, NMMO, and NLMO were synthesized via a typical solid-state method at 950 °C. All the precursors, including Na_2_CO_3_, LiOH, MgO, and Mn_2_O_3_, were purchased from Aladdin Co., Ltd., Shanghai, China. The precursors were mixed according to the appropriate stoichiometric ratios and then pressed into tablets. During the precursor preparation stage, we added 3 mol% excess Na_2_CO_3_ and LiOH·H_2_O to compensate for the loss of Na and Li volatilization at 950 °C. The samples were then calcined in a muffle furnace at 950 °C for 15 h with a heating rate of 4 °C min^−1^. After cooling to room temperature, the products were immediately transferred into an argon-filled glove box (H_2_O < 0.1 ppm, O_2_ < 0.1 ppm).

### 2.2. Material Characterization

All cathode materials were analyzed using X-ray diffraction (XRD, Japanese Rikagaku Ultima IV, Tokyo, Japan), scanning electron microscopy (SEM, JSM-7800F, Tokyo, Japan), transmission electron microscopy (TEM, JEM-2100Plus, Tokyo, Japan), energy-dispersive X-ray spectroscopy (EDS, connected to the JSM-7800F via an EDX detection system, Tokyo, Japan), and X-ray photoelectron spectroscopy (XPS, PHI5000 Versaprobe III XPS, Tokyo, Japan). The General Structure Analysis System (GSAS I, Revision 1251, Argonne National Laboratory, Lemont, IL, USA) was used to perform XRD Rietveld refinement to analyze the crystal structure. In situ X-ray diffraction measurements were performed using an electrochemical cell fitted with a beryllium window (Beijing Sijia Tuo Technology Co., Ltd., Beijing, China) on an Ultima IV powder X-ray diffractometer (Rigaku Corporation, Tokyo, Japan), and Cu Kα radiation (λ = 1.5406 Å) was utilized to collect diffraction patterns within the 2θ range of 10–50° at a scanning rate of 2°/min and a step size of 0.02°. The cell was cycled within the voltage range of 2.0–4.4 V under a constant current of 0.02 A/g. The cathode materials used for characterization after charge–discharge cycles were obtained by repeatedly rinsing them with diethylene glycol dimethyl ether in an argon-filled glove box and drying them in the glove box to remove solvents (H_2_O < 0.1 ppm, O_2_ < 0.1 ppm). For in situ XPS testing, the cathode material was first placed in the vacuum transition chamber of the glove box and then transferred into the ultra-high vacuum environment of the XPS.

### 2.3. Electrochemical Tests

Cathode electrodes were fabricated by mixing the active material (70 wt%), Super P carbon black (20 wt%), and polyvinylidene difluoride (10 wt%) in N-methyl-2-pyrrolidone (NMP). The resulting slurry was coated onto aluminum foil and dried at 110 °C for 12 h in a vacuum oven. The active material loading on the positive electrode was approximately 1.5 mg cm^−2^. Electrochemical tests were conducted using 2032-type coin cells with sodium metal as the counter electrode and 100 μL of 1 M NaPF_6_ dissolved in diethylene glycol dimethyl ether (DIGLYME, 100 vol%) as the electrolyte. Glass-fiber filters (Whatman GF/D) were used as separators. Constant-current charge/discharge and GITT measurements were performed on a battery test system (BTS-4000, NEWARE, Shenzhen, China). For GITT, a pulsed current of 20 mA g^−1^ was applied, with a pulse duration of 30 min and a relaxation time of 1 h. EIS was carried out on an electrochemical workstation (CHI-660E) with an AC amplitude of 10 mV in the frequency range from 100 kHz to 0.01 Hz.

### 2.4. Calculations of Na^+^ Diffusion Coefficients

The diffusion coefficient of Na^+^ ($D_{{Na}^{+}}$) was calculated using Formula (1).

(1) $$D_{{Na}^{+}}^{GITT}=\frac{4}{\pi \tau }\times {\left(\frac{m_{B}{\;V}_{M}}{M_{B}\;S}\right)}^{2}\times {\left(\frac{{∆E}_{S}}{{∆E}_{\tau }}\right)}^{2}$$

Here, τ represents the constant-current pulse time, and m_B_, V_M_, M_B_, and S respectively denote the mass of the anode material, molar volume, molar mass, and the area of the electrode/electrolyte interface. ${∆E}_{S}$ indicates the single-step voltage change, including the relaxation process, while ${∆E}_{\tau }$ refers to the battery voltage difference after subtracting the IR drop during the constant-current pulse step.

## 3. Results and Discussion

To determine the optimal Li/Mg doping ratio, we prepared a series of samples of Na_0.8_Li_x_Mg_0.3−x_Mn_0.7_O_2_ (x = 0, 0.06, 0.08, 0.10, 0.12, 0.3) and conducted their characterization (Figure S1). The XRD results indicated that all samples had a typical P2-type layered structure (Figure S1a), and the superstructure peak at 22.5° confirmed the successful doping of Li/Mg into the lattice, with only trace Li_2_MnO_3_ impurities (at 19.5°). The comparison revealed that Na_0.8_Li_0.08_Mg_0.22_Mn_0.7_O_2_ (NLMMO) exhibited the highest reversible capacity and the best cycle stability. Based on the systematic component results, we ultimately selected a doping ratio of Li = 0.08 and Mg = 0.22 as the target composition for this study and used the single-doped Na_0.8_Li_0.3_Mn_0.7_O_2_ (NLMO) and Na_0.8_Mg_0.3_Mn_0.7_O_2_ (NMMO) as comparison samples to further explore the synergistic modification mechanism of Li/Mg co-doping.

**Figure 1:**
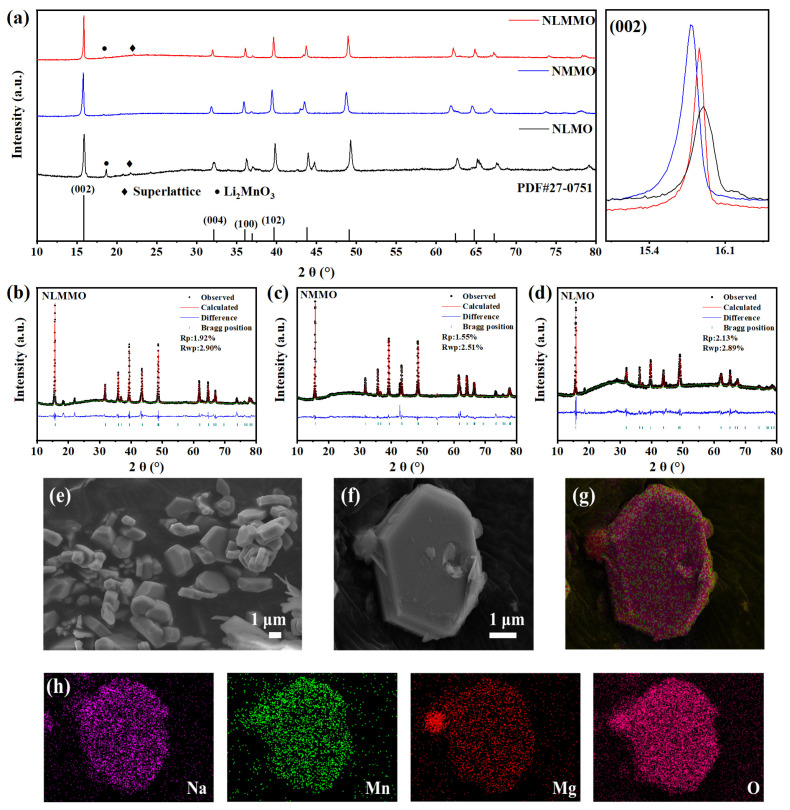
Structural characterization. (**a**) NLMMO, NMMO, and NLMO XRD curve comparison. (**b**–**d**) Rietveld refinements of the XRD patterns for NLMMO, NMMO and NLMO, respectively. (**e**,**f**) Scanning electron microscope images of the NLMMO samples and (**g**,**h**) the associated EDS elemental mapping results.

P2-Na_0.8_Li_0.08_Mg_0.22_Mn_0.7_O_2_ (NLMMO), P2-Na_0.8_Mg_0.3_Mn_0.7_O_2_ (NMMO), and P2-Na_0.8_Li_0.3_Mn_0.7_O_2_ (NLMO) were synthesized via the conventional solid-state reaction method. The X-ray diffraction (XRD) patterns of pristine Na_0.8_Li_0.08_Mg_0.22_Mn_0.7_O_2_ (NLMMO), Na_0.8_Mg_0.3_Mn_0.7_O_2_ (NMMO), and Na_0.8_Li_0.3_Mn_0.7_O_2_ (NLMO) are presented in Figure 1a. All the samples exhibit characteristic diffraction peaks corresponding to the P2-type layered structure with a hexagonal symmetry (space group: P6_3_/mmc), as confirmed by Rietveld refinement analysis. Notably, the (002) diffraction peak for NLMMO demonstrates an intermediate position between NMMO and NLMO, suggesting a systematic lattice parameter variation (Δc = 0.09 Å) induced by Li^+^ doping. This observation aligns with Vegard’s law, indicating successful Li substitution within the transition metal layer.

The General Structure Analysis System (GSAS I, Revision 1251, Argonne National Laboratory, USA) software was employed to analyze the corresponding refined models of the X-ray diffraction (XRD) patterns, as depicted in Figure 1b–d. XRD confirmed that all cathodes have a rhombohedral phase with space group P6_3_/mmc [33,34,35]. The crystal parameters in Tables S2–S4 show that the lattice spacings a, c of NLMMO confirm the successful doping of lithium and magnesium ions into the transition metal layer. Refined data analysis clearly shows that 0.08 mol of lithium ions replace manganese ions within the crystal lattice of NLMMO. A comparative evaluation of the XRD patterns of the samples shows that the characteristic peak of the (002) plane in NLMMO is positioned between those of NLMO and NMMO. This provides compelling evidence for the successful integration of lithium element into the sample matrix, implying a change in the crystal structure.

In Figure 1b, the superlattice peaks at 22.5° are clearly observed for NLMMO and NLMO. These peaks are likely attributable to the ordered distribution of lithium and manganese inside the transition metal (TM) layer, which adopts a $\sqrt{3}a\times \sqrt{3}a$ superlattice configuration, often referred to as the “1/3 1/3 1” superlattice [6,34,36]; the remaining weak peak (19.5°) corresponds to Li_2_MnO_3_, which does not affect the main structure or the electrochemical properties [9]. It should be noted that, compared to manganese, magnesium and oxygen, the X-ray scattering intensity of lithium is very low. Consequently, the lithium occupancy in the transition metal layer cannot be reliably refined. Consequently, in accordance with standard methods for lithium-containing layered oxide cathodes [6,22,28], the lithium occupancy was fixed at nominal values (0.08 for NLMMO and 0.30 for NLMO) based on the stoichiometric ratios of the precursors. Subsequently, under the constraint that the total transition metal occupancy sums to 1, the occupancy values of Mg and Mn were allowed to be adjusted. The consistency between the experimental and calculated XRD patterns (R_wp_ < 5%) and the agreement with ICP-OES results (Table S1, actual Li content = 0.077 vs. theoretical value of 0.08) both support the validity of this approach.

The crystal structure of NLMMO was further analyzed by transmission electron microscopy (TEM) (Figure S2). The lattice fringe spacing corresponding to the (002) crystal plane was determined to be 0.55 nm [37]. The morphologies of NLMMO, NLMO, and NMMO were characterized using scanning electron microscopy (SEM) (Figure 1e–h and Figure S3). After Li^+^/Mg^2+^ co-doping, the surface of NLMMO particles became smoother and more uniform. Elemental distribution was analyzed by energy-dispersive X-ray spectroscopy (EDS), confirming that Na, Mg, Mn, and O were homogeneously dispersed throughout the material.

**Figure 2:**
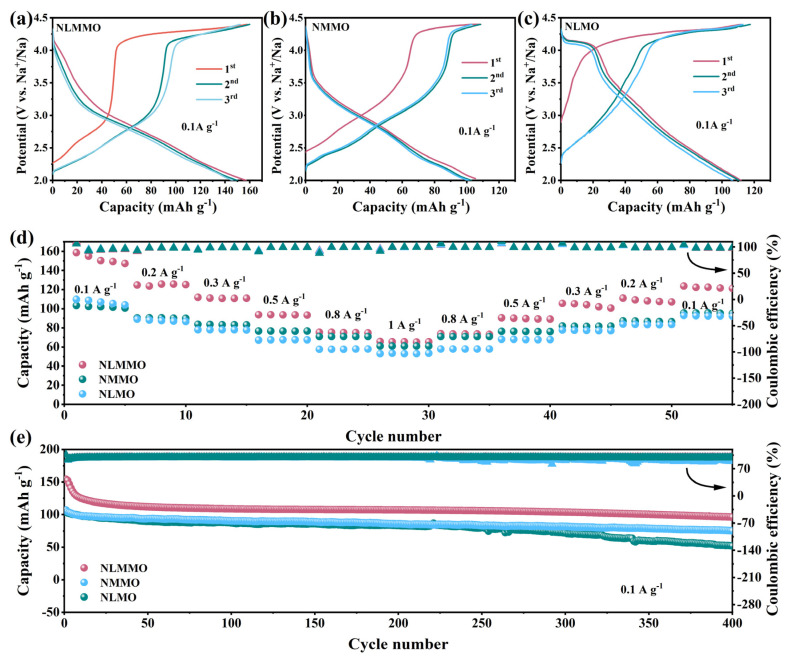
The electrochemical properties of NLMMO, NMMO and NLMO in the voltage range of 2.0–4.4 V. (**a**–**c**) Galvanostatic charge–discharge curves at 0.1 A g^−1^. (**d**) The rate performance and CE at a current density from 0.1 A g^−1^ to 1 A g^−1^. (**e**) Cycling durability was measured at a current density of 0.1 A g^−1^.

We then evaluated the electrochemical performance of the three cathodes in the voltage range of 2.0–4.4 V. Figure 2a–c present the galvanostatic charge/discharge profiles, which exhibit a two-stage charging behavior: an initial sloping region followed by a distinct voltage plateau above 4.2 V. The Coulombic efficiency of NLMMO (87.5%) is higher than that of NMMO (85.7%) and NLMO (83.3%) in the first cycle, and the polarization voltage (△V = V_ch_ − V_dis_) difference of NLMMO is lower than that of NMMO and NLMO for the same capacity.

Remarkably, NLMMO delivers the highest specific charge capacity of 158 mAh g^−1^, substantially exceeding those of NMMO (106 mAh g^−1^) and NLMO (110 mAh g^−1^). This indicates that Li/Mg co-doping enhances the charge storage capability of the cathode material. During discharge (involving reduction of both oxygen and manganese), the specific discharge capacity of NLMMO is higher than that of NMMO and NLMO; when NMMO doped with lithium, the charge plateau above 4.2 V is significantly enhanced and the slope of the discharge curve is significantly reduced. Due to the fact that Li ions can stimulate the oxidation–reduction of oxygen ions, these changes ultimately lead to a significant increase in capacity [9,28,38].

To comprehensively explore the differences in electrochemical performance between the cathodes, Figure 2d,e present a comparative analysis of life stability and rate performance with a voltage range of 2.0–4.4 V. Figure 2d clearly demonstrates that NLMMO achieves the best rate performance under varied current densities. The remarkable capacity retention exhibited by NLMMO provides compelling evidence of its robust stability. NLMMO exhibits reversible specific capacities of 158, 123, 118, 94, 87, and 77 mAh g^−1^ at current densities of 0.1, 0.2, 0.3, 0.5, 0.8, and 1 A g^−1^, respectively. Significantly, the capacity of NLMMO at a current density of 1 A g^−1^ is only 51% of that delivered at 0.1 A g^−1^. The initial discharge capacities of NLMMO, NMMO and NLMO are 158 mAh g^−1^, 106 mAh g^−1^ and 110 mAh g^−1^ at a current density of 0.1 A g^−1^. When doped with lithium and magnesium ions, the synergistic effect of lithium and magnesium co-doping reduces the proportion of trivalent manganese ions and mitigates the Jahn–Teller distortion [39]. However, excessive lithium and magnesium ions lead to poor structural stability and severe capacity loss28, [40,41,42]. As the number of cycles progresses, the NLMMO sample demonstrates a relatively high capacity retention rate. After 400 cycles, the NLMMO sample retains approximately 67% of its initial capacity. Although the capacity retention rate of the NMMO sample stands at 65%, it still outperforms the 50% capacity retention rate of the NLMO sample. This indicates that the specific doping strategy of NLMMO endows it with enhanced long cycling stability within the context of sodium ion battery applications.

**Figure 3:**
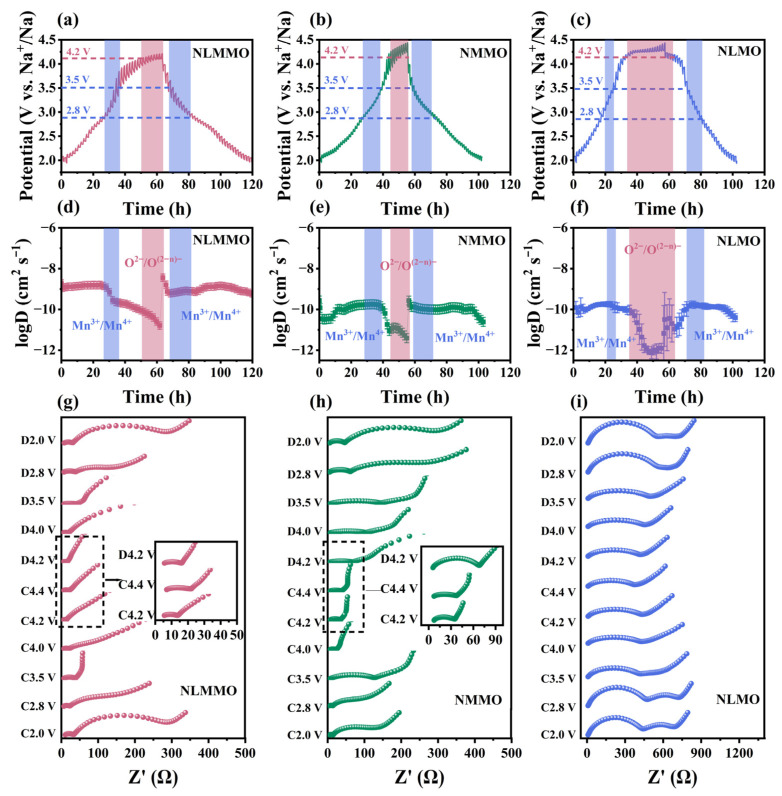
The electrochemical kinetics and sodium ion diffusion characteristics of the three samples. (**a**–**c**) GITT voltage curves of the first charge–discharge process for NLMMO, NMMO, and NLMO, with the corresponding oxidation–reduction potential intervals of Mn^3+^/Mn^4+^ cations (in blue background) and O^2−^/O^(2−*n*)−^ anions (in pink background) marked in the figure. (**d**–**f**) The corresponding sodium ion diffusion coefficients calculated from the GITT data, with error bars representing the standard deviations of three independent tests. (**g**–**i**) EIS Nyquist plots of NLMMO, NMMO, and NLMO at different charge–discharge states (2.0–4.4 V).

The Na^+^ diffusion coefficients ($D_{{Na}^{+}}$) were measured using the galvanostatic intermittent titration technique (GITT) (Figure 3). In the voltage range of 2.8–3.5 V, $D_{{Na}^{+}}$ decreases because the oxidation of Mn^3+^ causes a reduction in the unit cell parameter a and an increase in the Na^+^ diffusion barrier.

Between 4.2 and 4.4 V (Figure 3d–f), $D_{{Na}^{+}}$ drops sharply, likely due to oxygen-related redox reactions that induce c-axis contraction, thus affecting Na^+^ insertion and extraction. In the low-voltage regime (2.0–4.2 V), NLMMO and NLMO exhibit a diffusion level of 10^−9^ cm^2^ g^−1^, whereas NMMO demonstrates an order-of-magnitude-lower diffusivity (~10^−10^ cm^2^ g^−1^). Upon exceeding the critical voltage of 4.2 V, all the cathodes undergo an abrupt $D_{{Na}^{+}}$ attenuation, with NLMMO maintaining a remarkably higher diffusivity (1.71 × 10^−11^ cm^2^ g^−1^) than NMMO (6.92 × 10^−12^ cm^2^ g^−1^) and NLMO (1.88 × 10^−12^ cm^2^ g^−1^). This is attributed to the interleaved arrangement of Na-O-Mg and Na-O-Li structures, which improves the stability of the cathode under high pressure and enhances sodium ion transport kinetics.

Additionally, electrochemical impedance spectroscopy testing (EIS) was conducted on the cathodes under different voltage conditions (Figure 3g–i). The equivalent circuit diagram of EIS is shown in Figure S4. Table S5 shows the fitting results for the high-frequency semi-circle diameter, which represents the charge transfer resistance at the anode electrolyte/electrode interface. The resistance exhibited a decrease trend with increasing voltage (2 V–charged to 4.4 V). The lower R_ct_ of NMMO at C 2.0 V is an initial state effect. NLMMO exhibits much lower charge transfer resistance in the main working voltage range (≥3.5 V), leading to superior electrochemical performance. The doping of Li/Mg significantly reduced the charge transfer resistance, indicating an improvement in the electronic conductivity of the cathode after doping, with NLMMO exhibiting the highest electronic conductivity. As shown in the EIS results, the co-doping of Li and Mg effectively reduces the charge transfer resistance (R_ct_) during the cycling process.

**Figure 4:**
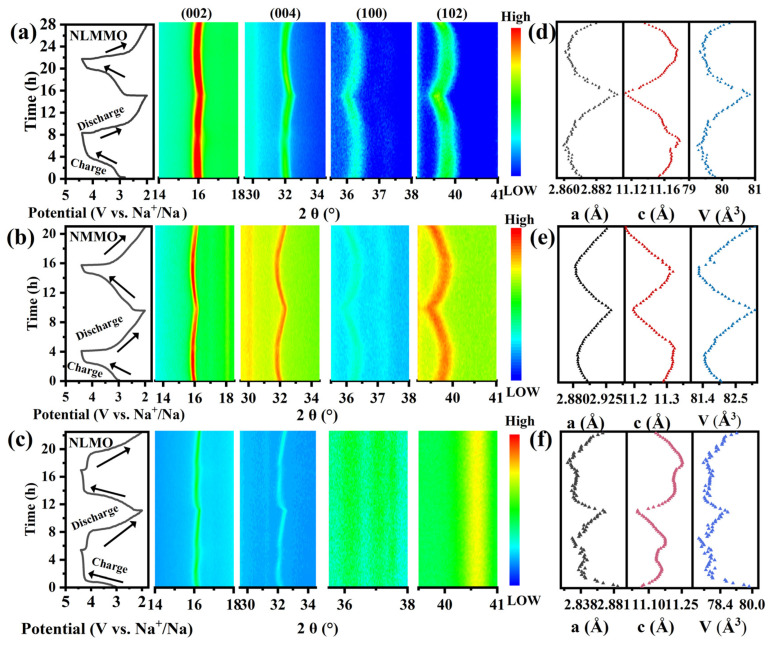
The structural evolution of NLMMO, NMMO, and NLMO during the cycling process. (**a**) The contour plot of the selected 2θ region in the in situ XRD pattern of NLMMO shows changes in the (002), (004) and (100) reflection peaks. (**b**) The contour plot of the selected 2θ region in the in situ XRD pattern of NMMO shows changes in the (002), (004) and (100) reflection peaks. (**c**) The contour plot of the selected 2θ region in the in situ XRD pattern of NLMO shows changes in the (002), (004) and (100) reflection peaks. (**d**–**f**) Changes in the unit cell parameters a/b (black dots), c (red dots) and V (blue dots) for the three samples NLMMO, NMMO and NLMO.

The electrochemical performance of the Mn-based sodium-ion layered oxide P2 phase depends on the stability and rapid transport of sodium ions. To demonstrate the effect of Li/Mg co-doping on the stability of the layered structure, in situ X-ray diffraction (XRD) measurements were performed on NLMMO, NMMO, and NLMO during the charging and discharging. In Figure 4a–c and Figure S5, the peaks marked with diamonds correspond to the Be window. The diffraction peaks of (002), (004), (100), and (102) can be clearly observed and attributed to P2 structure for NLMMO, NMMO, and NLMO cathodes. Both (002) and (004) peaks shift to the lower angles after charging, while the (100) and (102) peaks shift to higher angles. This is attributed to the increased electrostatic repulsion between the adjacent oxygen layers, leading to an increase in the lattice parameter c [36,43] and the oxidation of Mn^3+^ to a higher valence state due to Na^+^ deintercalation, resulting in a lattice contraction between the interlayer [44,45]. When the charging voltage exceeds 4.2 V, the (002) peak shifts slightly toward higher angles, indicating a weakened electrostatic repulsion due to the oxygen participation in the redox process. During discharge, as Na^+^ ions are intercalated, the diffraction peaks of both samples almost return to the original positions. Refined lattice parameters a and c for NLMMO, NMMO, and NLMO are shown in Figure 4d–f. NLMMO shows a smaller lattice parameter change (0.34%) compared to NMMO (1.71%) and NLMO (2.13%). The change in lattice parameter c (0.53%) is significantly smaller than that of NMMO (1.32%) and NLMO (1.51%). Within the 4.2–4.4 V voltage window, the change in lattice parameter c in NLMMO is 0.0017 Å, while it is 0.0006 Å for NMMO and 0.0043 Å for NLMO. Additionally, the unit volume change in NLMMO (1.76%) is smaller than that of NMMO (2.18%) and NLMO (2.43%), indicating that NLMMO may experience less irreversible oxygen loss, resulting in greater structural stability during charging and discharging. Furthermore, compared to NMMO and NLMO, the irreversible change in lattice parameter c after discharge to 2.0 V is significantly reduced for NLMMO, indicating improved structural reversibility and stability.

**Figure 5:**
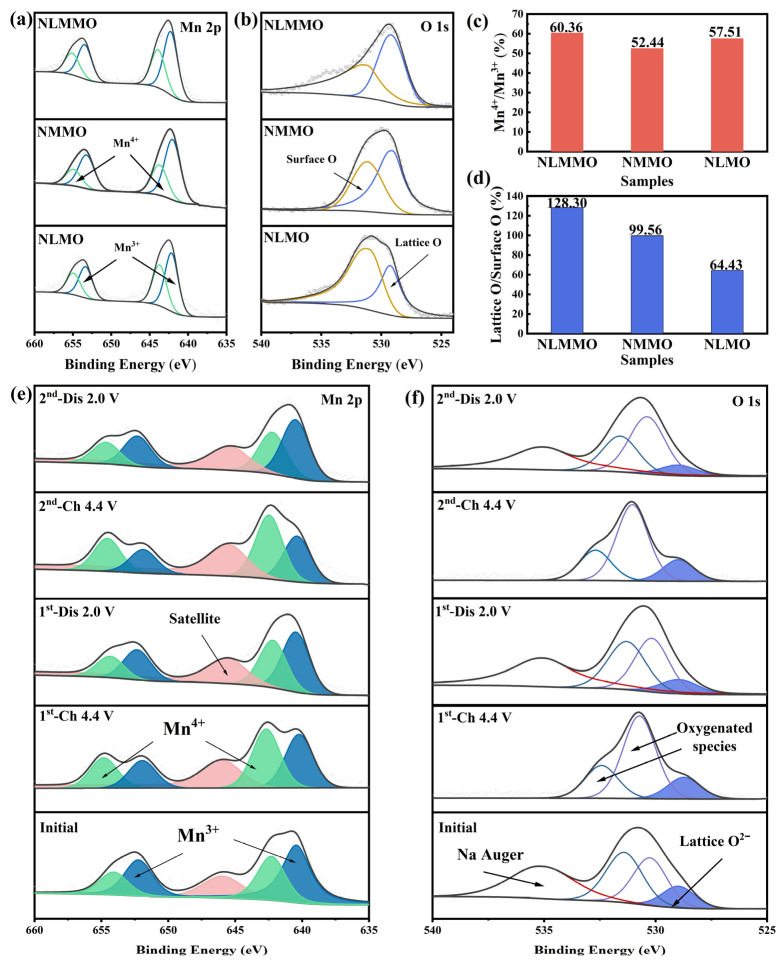
XPS spectra of the cathode sample surface: (**a**,**b**) Mn 2p and O 1s spectra of the three powder samples; (**c**,**d**) Mn^3+^/Mn^4+^ ratios and lattice oxygen/surface oxygen ratios of the powder samples; (**e**) XPS spectra of Mn 2p and (**f**) O 1s from the electrode sheets; these spectra were acquired from NLMMO electrodes in different charge–discharge states at a current density of 0.1 A g^−1^ within a voltage range of 2–4.4 V.

To elucidate the electronic states and bonding interactions of transition metals and oxygen on the surface of the cathode particles, we characterized the three samples using X-ray photoelectron spectroscopy (XPS). In Figure 5a, two main peaks corresponding to the Mn2p_3/2_ energy level are observed at 641.5 eV and 642.5 eV [42,46], indicating the presence of both Mn^3+^ and Mn^4+^ in the three initial samples. Further analysis of the Mn^3+^/Mn^4+^ ratio, as shown in Figure 5c, reveals that the proportions of Mn^3+^ are 60.36% for NLMMO, 52.44% for NMMO, and 57.51% for NLMO. These results demonstrate that Li doping in the NMMO sample leads to a reduction in Mn^3+^ content. It is widely acknowledged that the presence of Mn^3+^ in cathode materials can induce the Jahn–Teller effect, thereby compromising the stability of the layered structure. The synergistic effect of Li/Mg co-doping increases the Mn^4+^/Mn^3+^ ratio, reducing the Mn^3+^ content, mitigating the Jahn-Teller effect, and enhancing the structural stability of the samples.

In Figure 5b, two major peaks of the O1s energy level are observed at 529.7 eV and 530.8 eV [47], which correspond to lattice oxygen and surface oxygen, respectively. As compared in Figure 5d, the Li/Mg co-doped sample exhibits the highest lattice oxygen-to-surface oxygen ratio. With the increase in lattice oxygen content, the bonding energy between transition metals and oxygen is gradually strengthened. Under the synergistic effect of Li/Mg co-doping, an interwoven structure of Na-O-Mg and Na-O-Li is formed, which further enhances the stability of the layered structure.

For a comprehensive analysis of the charge compensation in NLMMO, X-ray photoelectron spectroscopy (XPS) analysis on different charge/discharge states was conducted, as shown in Figure 5e and Figure S6. The deconvoluted Mn 2p of NLMMO demonstrates that the Mn 2p_3/2_ and Mn 2p_1/2_ binding energies at 641.5 and 653 eV [48,49] correspond to Mn^3+^. Both the Mn2p_3/2_ and Mn2p_1/2_ peaks shift towards the higher binding energies when charging to a voltage of 4.4 V, indicating the emergence of the Mn^4+^ state with Mn 2p_3/2_ and Mn 2p_1/2_, located at 642.5 and 654 eV, respectively [49,50]. When discharged to 2.0 V, 43% of Mn^4+^ remains in the battery, but cycle stability is not affected. In the following charge cycle, 9% of Mn^3+^ is oxidized to Mn^4+^ and then reduced back to Mn^3+^ during discharge. The results suggest the synergistic effect of lithium and magnesium ions in the transition metal layer, which mitigates the Jahn-Teller effect. In order to demonstrate the anionic redox mechanism of lattice oxygen, XPS analyses of O 1s on NLMMO at different charge/discharge states were conducted (Figure 5f). The peak at 529.7 eV corresponds to the O^2−^ lattice [10,50]. The peaks with high binding energies (531.8, 532.6, and 536.4 eV) correspond to the surface matter and Na, respectively [48]. Upon charging to 4.4 V, a change in the relative intensity of the O 1s signal was observed at approximately 529.7 eV, corresponding to changes in the lattice oxygen within the electrode material. After two charge–discharge cycles to 4.4 V, the characteristic peaks of oxygen-related species in the O 1s spectrum shifted slightly towards higher binding energies. According to the latest guidelines for O 1s XPS analysis, this shift is attributed to changes in the surface chemical environment, including the formation of surface hydroxyl and carbonate groups during cycling, as well as the generation of electrolyte decomposition products, rather than redox reactions involving lattice oxygen. The reversible changes in these surface species confirm that the electrode surface maintains structural stability during cycling. These findings demonstrate that the Li/Mg co-doping strategy is an effective approach to mitigate lattice oxygen release in sodium-ion batteries and enhance the cyclability of oxygen redox.

## 4. Conclusions

In this work, we systematically compared the electrochemical performance of three cathode materials: NLMMO, NLMO, and NMMO. Li^+^ substitution strongly stimulates the anionic redox. Mg^2+^ doping can effectively support the transition metal (TM) layer, preventing interlayer slip caused by sodium ion deintercalation, thereby improving capacity retention. As a result, the P2-type NLMMO cathode delivers an initial specific discharge capacity of 158 mAh g^−1^ and retains 68% of that capacity after 400 cycles. In situ XRD confirmed that the NLMMO cathode has a stable structure during charging and discharging, and XPS revealed that the synergistic effect of Li/Mg co-doping enables reversible oxygen redox in NLMMO. This study reveals that in-depth exploration of the doping mechanism of doubly inert transition metals in layered oxide materials will provide more experimental and theoretical insights for the design of efficient cathode materials for sodium-ion batteries.

## Data Availability

The original contributions presented in this study are included in the article/supplementary material. Further inquiries can be directed to the corresponding author.

## Supplementary Materials

The following supporting information can be downloaded at: https://www.mdpi.com/article/10.3390/ma19102006/s1: Figure S1: Compare the structures and properties of the four samples; Figure S2: TEM image of NLMMO, which demonstrates a typical layer structure; Figure S3: Structural characterization. (a) SEM image of NLMO and the corresponding EDS mapping. (b) SEM image of NMMO and the corresponding EDS mapping; Figure S4: Equivalent circuit for fitting impedance spectra; Figure S5: The structural evolution of NLMMO, NMMO, and NLMO during the cycling process; Figure S6: Percentage between Mn^3+^ and Mn^4+^ calculated from 2p_3/2_ peak area fit spectra of Mn^3+^ and Mn^4+^; Table S1: ICP-OES results of as-prepared cathode materials. Table S2: Lattice parameters of P2-NLMMO material derived from Rietveld Refinement; Table S3. Lattice parameters of P2-NMMO material derived from Rietveld Refinement; Table S4. Lattice parameters of P2-NLMO material derived from Rietveld Refinement; Table S5. Diameters of semicircles in EIS plots of NLMMO, NMMO, NLMO. ICP-OES, TEM, EDS, in situ XRD, XPS, in situ EIS and tables.
