# Supplementary material for: Mg/Li Co-Doping Activates Anionic Redox in Sodium-Ion Battery Layered Oxides
Source: Materials (Basel). 2026 May 12;19(10):2006. doi: 10.3390/ma19102006 (PMC13208522; doi:10.3390/ma19102006)
Supplement: Supplementary file 1 [file materials-19-02006-s001.zip › materials-4275146-supplementary.pdf]

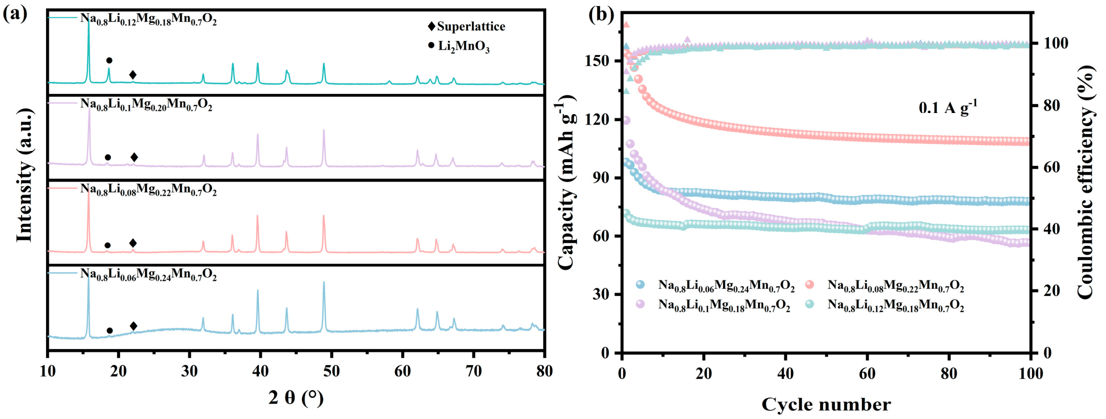

Figure S1. Compare the structures and properties of the four samples: $Na_{0.8}Li_{0.06}Mg_{0.24}Mn_{0.7}O_2$, $Na_{0.8}Li_{0.08}Mg_{0.22}Mn_{0.7}O_2$, $Na_{0.8}Li_{0.1}Mg_{0.20}Mn_{0.7}O_2$, $Na_{0.8}Li_{0.12}Mg_{0.18}Mn_{0.7}O_2$. (a) The XRD patterns of four samples with different Li/Mg doping ratios show a typical P2-type structure, which includes superlattice structures and trace amounts of Li_2MnO_3. (b) The cycling performance test conducted at a current density of 0.1 A g^{-1} indicates that the $Na_{0.8}Li_{0.08}Mg_{0.22}Mn_{0.7}O_2$ sample exhibits the best capacity and stability.

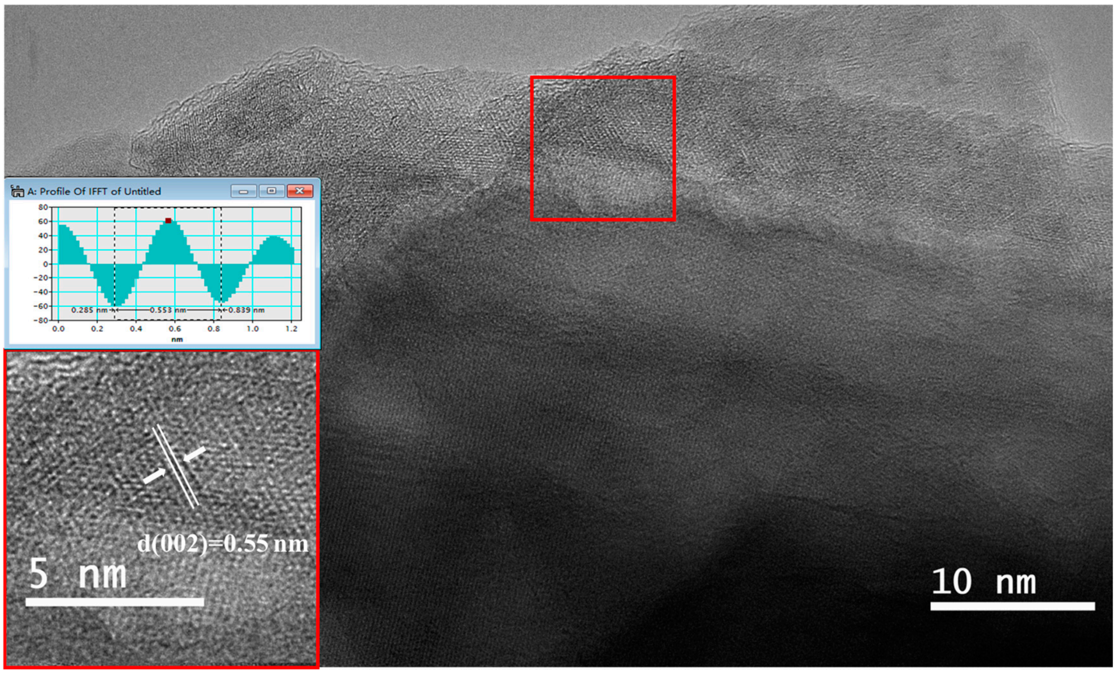

Figure S2. TEM image of NLMMO, which demonstrates a typical layer structure.

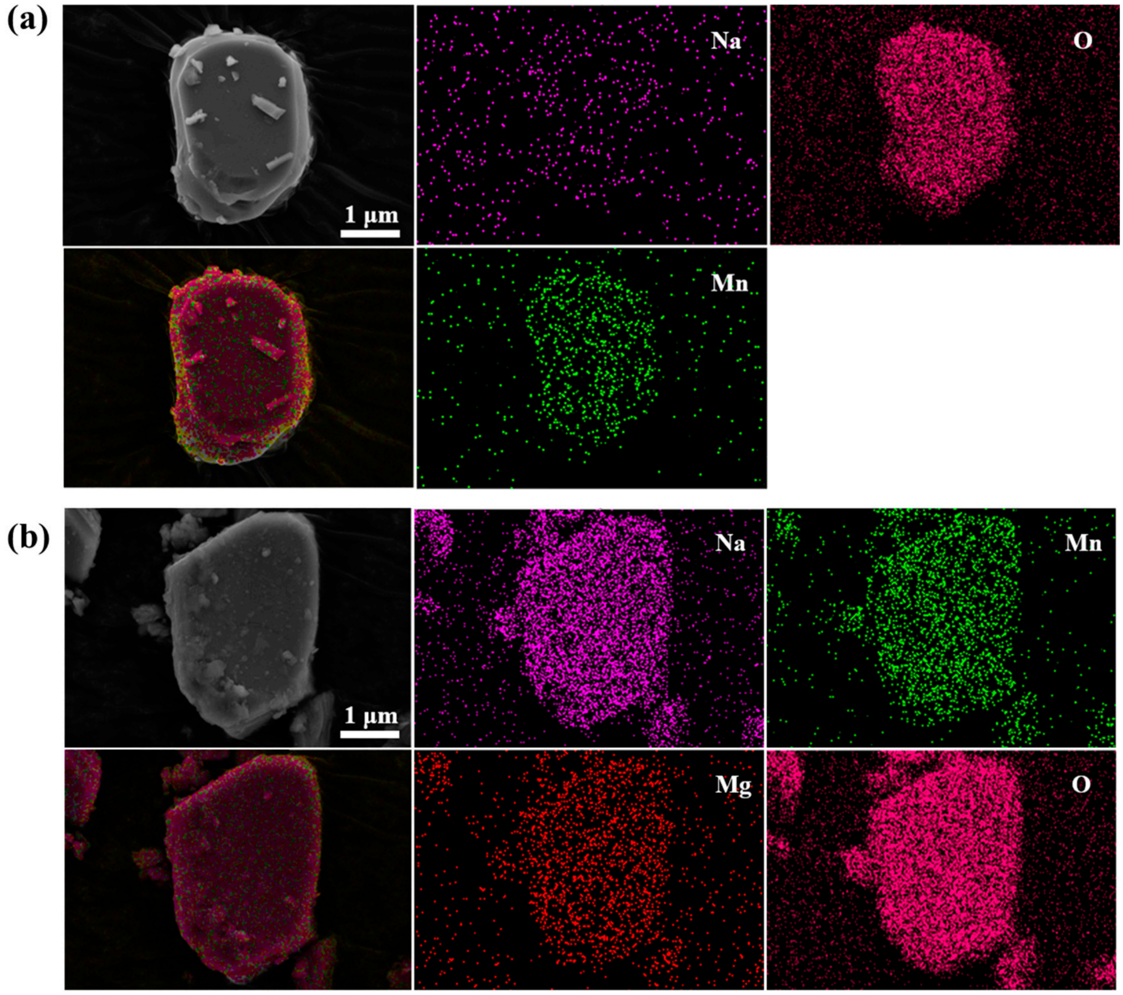

Figure S3. Structural characterization. (a) SEM image of NLMO and the corresponding EDS mapping. (b) SEM image of NMMO and the corresponding EDS mapping.

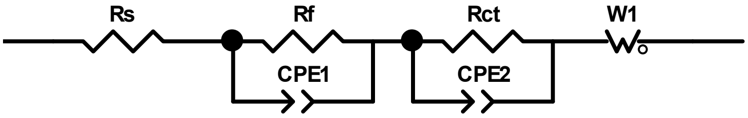

Figure S4. Equivalent circuit for fitting impedance spectra.

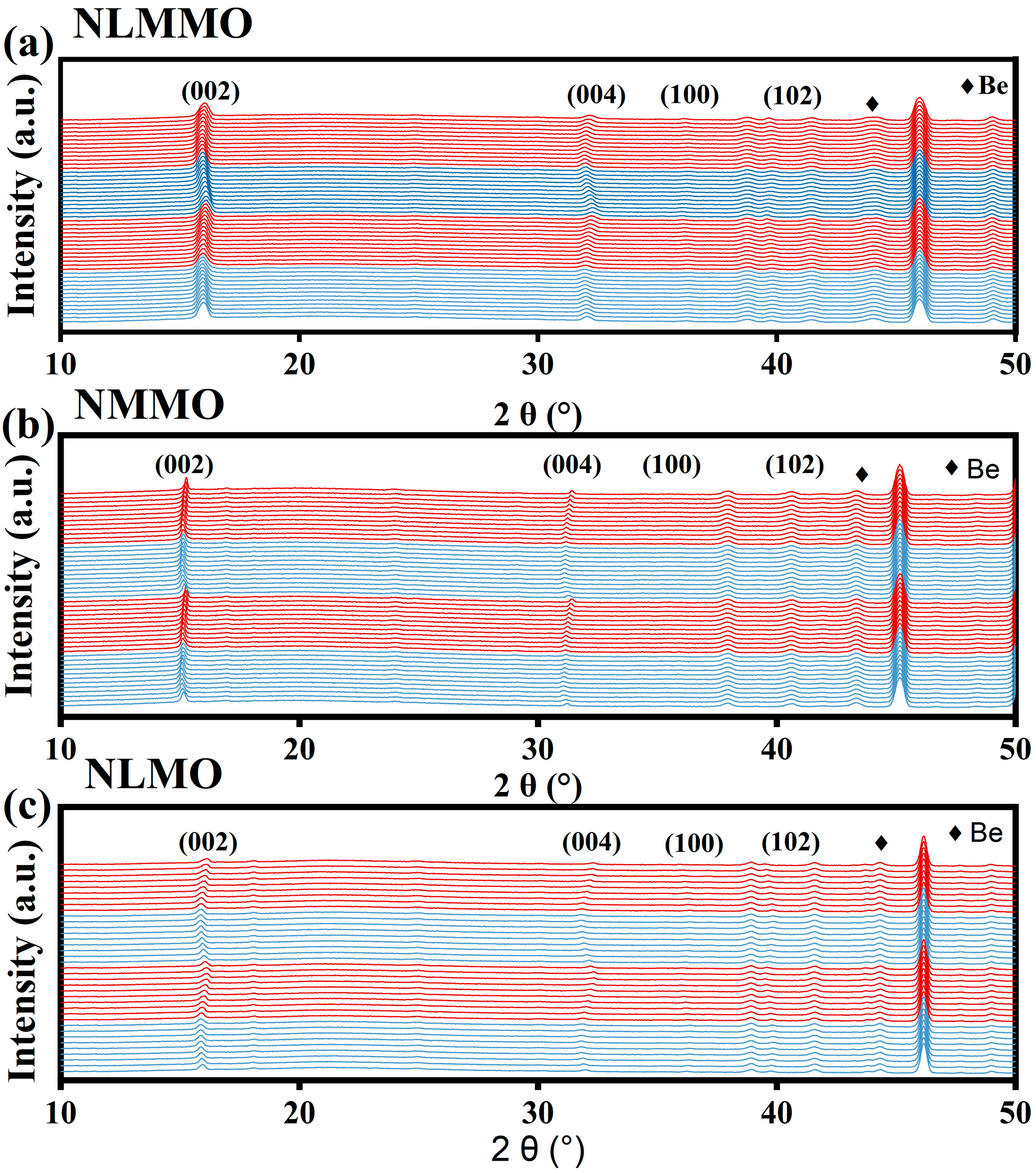

Figure S5. The structural evolution of NLMMO, NMMO, and NLMO during the cycling process. (a) The typical in situ XRD patterns of NLMMO electrode at 0.02 A g^{-1} in the voltage window of 2.0–4.4 V. (b) The typical in situ XRD patterns of NMMO electrode at 0.02 A g^{-1} in the voltage window of 2.0–4.4 V. (c) The typical in situ XRD patterns of NLMO electrode at 0.02 A g^{-1} in the voltage of 2.0–4.4 V.

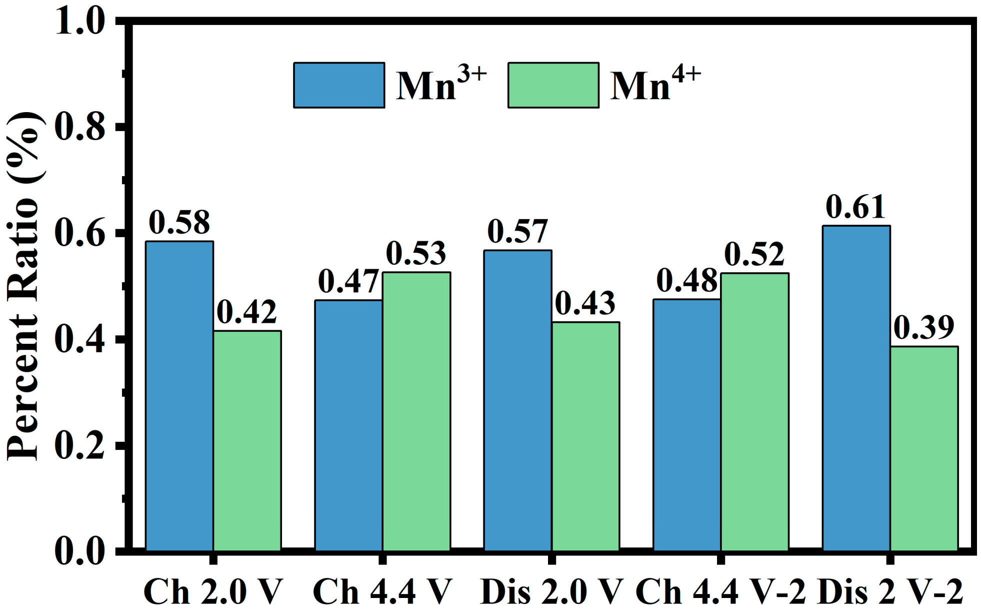

Figure S6. Percentage between Mn^{3+} and Mn^{4+} calculated from $2p_{3/2}$ peak area fit spectra of Mn^{3+} and Mn^{4+}.

Table S1. ICP-OES results of as-prepared cathode materials.

Sample	Measured atomic ratio			
	Na	Li	Mg	Mn
$Na_{0.8}Li_{0.08}Mg_{0.22}Mn_{0.7}O_2$	0.789	0.077	0.215	0.684

Table S2. Lattice parameters of P2-NLMMO material derived from Rietveld Refinement.

Atom	site	a	b	c	occ
Na_e	2d	0.6667	0.3333	0.2500	0.4361(4)
Na_f	2f	0.0000	0.0000	0.2500	0.3679(4)
Mn_1	2a	0.0000	0.0000	0.0000	0.7000(1)
Li_1	2a	0.0000	0.0000	0.0000	0.0800
Mg_1	2a	0.0000	0.0000	0.0000	0.2200(5)
O_1	4f	0.6667	0.3333	0.0917	1.0000

Space group: $P6_3/mmc$ Rp:1.92%, Rwp:2.90%, chi^2 :8.823. a=b=2.880(2), c=11.251(3).

Table S3. Lattice parameters of P2-NMMO material derived from Rietveld Refinement.

Atom	site	a	b	c	occ
Na_e	2d	0.6667	0.3333	0.2500	0.4372(4)
Na_f	2f	0.0000	0.0000	0.2500	0.3668(4)
Mn_1	2a	0.0000	0.0000	0.0000	0.7000(3)
Mg_1	2a	0.0000	0.0000	0.0000	0.3000(5)
O_1	4f	0.6667	0.3333	0.0728	1.0000

Space group: $P6_3/mmc$ Rp:1.55%, Rwp:2.51%, chi^2 :5.517. a=b=2.900(3), c=11.260(4).

Table S4. Lattice parameters of P2-NLMO material derived from Rietveld Refinement.

Atom	site	a	b	c	occ
Na_e	2d	0.6667	0.3333	0.2500	0.4358(3)
Na_f	2f	0.0000	0.0000	0.2500	0.3682(3)
Mn_1	2a	0.0000	0.0000	0.0000	0.7600(2)
Li_1	2a	0.0000	0.0000	0.0000	0.3000
O_1	4f	0.6667	0.3333	0.0924	1.0000

Space group: $P6_3/mmc$ Rp:2.13%, Rwp:2.89%, chi^2 :3.193. a=b=2.868(1), c=11.216(9).

Table S5. Diameters of semicircles in EIS plots of NLMMO, NMMO, NLMO.

Voltage region	NLMMO	NMMO	NLMO
C2.0 V	194.3 Ω	78.7 Ω	406.8 Ω
C2.8 V	86.1 Ω	43.2 Ω	439.3 Ω
C3.5 V	29.4 Ω	47.3 Ω	378.2 Ω
C4.0 V	27.6 Ω	29.4 Ω	369.9 Ω
C4.2 V	15.3 Ω	36.1 Ω	383.9 Ω
C4.4 V	22.3 Ω	36.5 Ω	336.6 Ω
D4.2 V	16.8 Ω	62.3 Ω	378.1 Ω
D4.0 V	25.1 Ω	130.1 Ω	409.3 Ω
D3.5 V	29.9 Ω	150.4 Ω	442.8 Ω
D2.8 V	73.9 Ω	167.7 Ω	477.5 Ω
D2.0 V	194.7 Ω	195.2 Ω	470.2 Ω
